# MSML-DenseXmer: harnessing vision transformers through integration with novel dense networks for medical image fusion

**DOI:** 10.1038/s41598-026-52181-8

**Published:** 2026-05-29

**Authors:** Disha Mohini Pathak, Dhyanendra Jain, Somya Srivastava, Rajendra Kachhava, Tribhuwan Kumar Tewari, Surbhi Sharma

**Affiliations:** 1 Department of Computer Science and Engineering, ABES Engineering College, Ghaziabad, Uttar Pradesh India; 2 Department of CSE-AIML, ABES Engineering College, Ghaziabad, Uttar Pradesh India; 3 Computer Science Department, Indian Institute of Information Technology Kota, Kota, Rajasthan India; 4 CSE and IT Department, Jaypee Institute of Information Technology, Noida, Uttar Pradesh India; 5 Department of Computer Science and Engineering, Manipal University Jaipur, Jaipur, 303007 Rajasthan India

**Keywords:** Computational biology and bioinformatics, Engineering, Health care, Mathematics and computing, Medical research

## Abstract

The integration of multiple modalities in medical imaging allows a thorough representation of structural and functional details, resulting in improved diagnosis and treatment. Deep learning methods outperform conventional methods by automating the extraction of pertinent features and fusing them while preserving both structural and textural integrity. Existing methods lack the ability to capture complex global structures, small-scale textural features, and long-range dependencies, which causes incomplete feature representation. This study presents a novel deep-learning framework that combines an improved DenseNet for capturing local fine- grained features with a Swin Transformer for extracting global structural details and long- range relationships, thereby facilitating a more comprehensive fused output. A modified hybrid approach utilizing L1, L2 and infinity norm is used to generate attention weights in the feature fusion-oriented row-column vector dimension technique. The model is trained on different modalities in the Whole Brain Atlas dataset and the Lung-PET-CT-Dx dataset using a novel loss function. This function improves fusion by integrating pixel loss, structural similarity, and textural preservation. The evaluation of the fused image’s quality involves multiple metrics that assess image clarity, structural integrity, feature retention, contrast improvement, and overall visual accuracy, providing a thorough analysis. The MSML-DenseXmer framework demonstrates improved performance compared to existing approaches across multiple medical imaging modalities. Specifically, it achieves over 9.94% rise in MRI-SPECT fusion, above 6.82% gain in MRI-PET fusion, a minimum of 18.37% increase in MRI-CT fusion, and at least 2.83% gain on the Lungs PET-CT dataset, indicating its potential in improving fusion quality.

## Introduction

Modern healthcare has highly utilized medical imaging, and each diagnostic method has its own beneficial diagnostic data, which include MRI, CT, PET, and SPECT. MRI is of superior quality of soft tissue contrast, CT is accurate in recording bone structure, PET shows metabolic processes, whereas SPECT is able to show blood flow and metabolic activity by detecting gamma rays^1^^,2^. Nonetheless, the individual modality is incapable of providing all structural and functional information needed to make accurate diagnosis. Medical image fusion tackles this shortcoming by integrating the pertinent data of various modalities into a single composite image, which helps show the anatomical features, functional and metabolic activity at the same time^3^. This is particularly useful in cancer therapy, neurology, and radiology whereby a multi-modal information is vital to informed clinical decisions.

In the time span of 10–15 years, several methods for integrating clinical images have been proposed, mostly classified into traditional methods and methods based on deep learning. Traditional techniques, such as Principal Component Analysis (PCA)^4^ spatial domain^5^, discrete wavelet transform (DWT)^6^^,7^^,8^^,9^^,10^^,11^^,3^ dual-tree discrete wavelet transform (DTDWT)^12^, dual-tree complex wavelet transform (DTCWT)^13^^,14^, non-subsampled contourlet transform (NSCT)^15^^,13^^,16^, non-subsampled shearlet transform (NSST)^14^^,17^^18^, ripplet transform (RT)^19^, stationary wavelet transform (SWT)^11,^^20^ and Pulse Coupled Neural Network (PCNN)^17^^,16^ apply mathematical transformations to combine images. However, these methods typically do not effectively retain important details and face challenges in achieving a balance between contrast and sharpness. These approaches provide limited flexibility in handling intricate features and are susceptible to losing crucial information from the original modality while performing fusion.

One way that deep learning methodologies have overcome these limitations is by automatically learning more complex features during convolutional processes by extracting both low-level (edges, textures) and higher-level features (forms, structures) without human input^21^; (Liu, Chen, Cheng, et al., 2017)^1^;. Nevertheless, basic CNN models are not able to capture long-range interdependencies because convolutional operations are local for example, CNNs are very good at local feature extraction, but lack global structural information and multi scale relationships. In order to bridge this gap, this paper proposes the MSML-DenseXmer, a novel deep learning network to fuse medical images, which combines an innovative Multi-Scale Multi-Level Dense Block with a Swin Transformer^22^^,23^^,24^, to extract both local and global features simultaneously. The main contributions of this study are:

1. Three cascaded dense blocks with successively growing kernel sizes (3, 5, and 7) and filter counts (32, 64, and 128) are suggested forming a novel Multi-Scale and Multi-Level Dense Block (MSML-Dense Block) and an extension of the standard DenseNet architecture. Compared to traditional DenseNet where it uses a single scale with identical kernels, the MSML-Dense Block simultaneously extracts fine-grained textures, intermediate structural patterns, and large-scale contextual patterns by its multi-scale structure. This block is combined with the Swin Transformer into a two-branch design, with local multi-scale feature extraction being done with the MSML-Dense Block and the global high-level dependency being learned with the Swin Transformer via shifted window-based self-attention. This complementary dual-branch design has not been previously explored for medical image fusion. Current fusion techniques either use CNNs alone (DenseFuse, IFCNN, U2Fusion) which do not give global context, or use transformers alone, which can miss fine- grained local textures.
2. An innovative hybrid fusion attention mechanism is proposed to feature fusion, involving the L1 norm, L2 norm, and Infinity norm in a row-column vector dimension framework. This particular mixture is not suggested in previous fusion literature. The L1 norm represents the distribution of intensity, the L2 norm is the feature energy, and the Infinity norm is the salient features. The mechanism is able to deal with the heterogeneous nature of feature characteristics, which single-norm approaches fail to deal with, by combining all three norms with learnable weights. The two-dimensional (row and column) attention also makes sure that the spatial dependencies are accounted along both dimensions giving less coarse control over the integration of features than traditional channel-wise or spatial-only attention systems.
3. It introduces a new composite loss function that combines pixel loss (MAE), structural similarity loss (SSIM) and Gray Level Co-occurrence Matrix (GLCM) loss. Although the pixel and SSIM loss merging is typical in image reconstruction activities, the use of GLCM loss to maintain texture when a medical image undergoes a fusion activity is new. GLCM loss is an explicit preservation of spatial co-occurrence statistics between pairs of pixels which preserve diagnostically important textural features including tissue homogeneity and micro-structural irregularities not preserved by pixel-level and structural losses. This tri-component loss fills a gap in current fusion models that would generally optimize accuracy, structural consistency of pixels, but do not explicitly model textural fidelity.

Although the elements of individual components of the proposed framework would be inspired by the existing architectures, their specific integration constitutes a novel and synergistic design that has not been previously explored. None of the current medical image fusion designs implements a dual-branch architecture with multi-scale dense connectivity with shifted window-based transformers, a triple-norm hybrid attention to fusion between row and column vectors of dimension, and a texture-aware GLCM loss. The originality of the work is not in the separate building blocks, but in the specific purpose for which they are combined to address the challenge of simultaneously preserving local textures, global structures, and textural fidelity in multi-modal medical image fusion that none of the existing methods can directly meet the need.

The subsequent portions of the paper are structured as follows. Section 2 contains the literature review, followed by the methodologies in Sect. 3. Section 4 presents the implementation specifics. This study delineates the performance evaluation, and results and discussion in Sect. 5, and 6, respectively. Section 7 delineates the relevant conclusion.

## Literature review

Medical image fusion can be achieved by pixel fusion, feature fusion, or decision fusion^2^; (Singh, Singh, Bueno, et al.^25^,). The conventional method consists of three steps: breakdown of images into basic parts, a fusion rule, and the reconstruction of a composite image^3^. Current methods can be classified into five groups (1) traditional methods based on spatial and frequency domains, (2) sparse representation methods, (3) hybrid methods that are based on both traditional and deep learning methods, (4) supervised deep learning methods, and (5) unsupervised deep learning methods. Table 1 gives an in-depth description of these methods and their challenges.

Deep learning has greatly improved the process of fusing medical images by automating the extraction and fusion of features^1^^,37^. Dao et al.^38^, addresses the modality uncertainty by introducing inter-modality feature fusion which is further extended by^39^, introduced inter and intra modality feature fusion using AttCo framework for adaptive feature representation. Although deep learning approaches perform better than traditional methods, they have drawbacks such as a high reliance on huge datasets, lengthy processing times, significant memory needs, and challenges in managing long-term dependencies. To overcome this limitation^40^incorporate attention mechanism with different CNN models like VGG16, ResNet18 and EfficientNet to enhance the feature representation and further utilize those features for brain tumor classification. Transformers have been integrated with machine learning and deep learning techniques to enhance the ability to perform fusion. Ullah and Kim^41^, perform feature fusion using pre-trained vision transformer to further classify it using machine learning classifier. Since the quality of image fusion largely depends on how well features are represented, recent studies have increasingly focused on improving the extraction of both local details and global contextual information. For better feature representation researchers performed feature learning using transformers to work better in low visibility scenarios. The key to enhancing output quality is to improve feature representation. Hussain et al.^42^, utilized hierarchical encoding with multi scale cross attention to enhance feature representation by introducing transformer-based framework. Cao et al.^43,44^, focus on feature representation using attention mechanism to enhance the outcome. In light of these developments, it is imperative to concentrate on enhancing the quality and interpretability of the fused output by extracting both local and global features from medical pictures and creating an effective fusion approach that maintains all important details.

**Table 1:** Summary of related research: conventional and deep learning-based approaches for image fusion.

Paper	Category	Method	Dataset	Quality metric	Challenges
Li et al.^5^,	Conventional (Spatial domain)	The image is first decomposed on two scales, then fused using weighted averaging based on guided filtering, and then reconstructed using two-scale image reconstruction.	Petrovic image database	Mutual information, Structural similarity, universal image quality index, entropy	Highly computational, manual intervention, pixel irregularities, image distortion, processing time is high, lacks fine details, low contrast, and no multi-scale information.
He et al.^4^,	Conventional (Spatial domain)	Decomposition utilizing HIS and fusion using PCA by replacing new intensity by the intensity of PET	AANLIB	Mutual information	low edge retention, noise sensitivity, inability to capture fine details, pixel irregularities, and image distortion.
Ramaraj et al.^9^,	Conventional (Frequency domain)	DWT decomposition is used, and the weighted average of the high frequency component is used for fusion. The approximation component is accepted exactly as it is.	Brain data (Kag- gle)	Fusion Factor, Mean square error, peak signal to noise ratio, structure similarity	Manual process, pixel based fusion, low contrast images.
Alzahrani^6^,	Conventional (frequency domain)	Decomposed using modified DWT and fusion is performed between approximate details of one source image and high frequency details of another image.	AANLIB	Feature Fusion, Entropy, Structure similarity	shift invariant, manual process, low contrast, lags in extracting fine details.
Aghamaleki and Ghorbani^12^,	Conventional (frequency domain)	Decomposed using DTDWT into 6 levels and fusion is performed using weighted average rule and weight are optimized by maximizing entropy and minimizing mean square error.	TNO Database	root mean square error, structural similarity, Entropy	manual process, doesn’t work in all direction, low contrast, loss of fine details, lags in multi scale features.
Babu and Narayana^14^,	Conventional (frequency domain)	Decomposed at two stages using DTCWT and NSST. Fused using fuzzy logic and max rule at stage 1 and average rule and deep neural network at stage 2.	AANLIB	Mutual information, root mean square error, structural similarity, standard deviation, entropy, peak signal-to-noise ratio.	manual process, High computational cost, low contrast, loss of fine details, processing time is high.
Singh et al.^3^,	Conventional (frequency domain)	Decomposition is accomplished with DWT. Bilateral filter is applied to determine the weights. Weighted averaging is then used to integrate these high-frequency coefficients, whereas simple averaging is used to merge low-frequency components.	TNO Dataset	Sum of correlation difference, artifact measure, edge-based similarity index, structural similarity.	shift invariant, not a generalize method, lack complex details, low contrast, distorted output.
Ibrahim et al.^16^,	Conventional (frequency domain)	Source images are broken down by NSCT into low- and high-frequency sub-bands, which are then combined using PCNN. After that, inverse NSCT is utilized to recreate the merged image.	AANLIB	Standard deviation, weighted edge information, peak signal-to-noise ratio, mutual information, average gradient, entropy.	manual tuning of parameters, High computational cost, blurring artefact on smoothing, incapable for intricate details.
Ch et al.^15^,	Conventional (frequency domain)	Input images are broken down by NSCT and fused using mean filter and least squares filter.	Private Hospi- tal Data reposi- tory	Edge quality index, feature mutual information, edge contribution, similarity index, mutual information.	human driven process, high computational cost, Contrast issue, preprocessing and pre-registration is missing.
Alseelawi et al.^13^,	Conventional (frequency domain)	Use NSCT and DTCWT to break down source images into low and high frequency coefficients. Determine the absolute deviations and thresholds for fusion, then choose the coefficients that deviate the most. Lastly, use INSCT and IDTCWT to recreate the fused image.	AANLIB	Visual information fidelity, fusion factor, peak signal-to-noise ratio, fusion symmetry.	No quality control, shift invariant, High computational cost, incapable for intricate details.
Diwakar^11^,	Conventional (frequency domain)	Decomposition using DWT and SWT and fused using pixel wise sum and reconstruction using respective inverse transforms	Brain scan dataset	Mean square error, entropy, peak signal to noise ratio, variance, mutual information, standard deviation.	No Directional data, data loss, manual process. tiny details of image are missing.
Preethi and Aishwarya^10^,	Conventional (frequency domain)	DWT is utilized for decomposition, weighted average rule and pixel-wise sum are used for fusion, and inverse transform is used for reconstruction.	AANLIB	Not available	Shift invariant, ring artefacts, noise, manual process, and incapable to extract complex traits.
Bhardwaj and Nayak^8^,	Conventional (frequency domain)	Decomposed using DWT and fusion is performed using Bayesian fusion optimized using bird swarm optimization.	BRATS Database	root mean square error, peak signal-to-noise ratio, Mutual information	manual process, shift invariant, low contrast, pixel level fusion.
Singh and Anand^19^,	Conventional (frequency domain)	Following input image decomposition by the RT, fusion is carried out utilizing the sum modified Laplacian, maximum rule, and sum modified spatial frequency. The Inverse RT is used to recreate the merged image.	AANLIB	Spatial frequency, entropy, edge index, standard deviation, and mutual information.	A distinct module for handling noise and performance makes the system computationally demanding, distort the fine details.
Barani and Sumathi^7^,	Conventional (frequency domain)	Decomposition using DWT and approximate coefficients fused using Adaptive weighted average fusion rule and reconstruction using inverse DWT.	Rider Lung PET/CT (TCIA)	Entropy, Spatial Frequency, Fusion Factor, Mutual Information, Standard Deviation	lags in fusing detail coefficients, Low contrast, image distortion, and low edge retention.
Jana and Das^26^,	Conventional (frequency domain) and sparse repre- sentation	A two-stage decomposition is utilized. The first stage uses fuzzy c mean and second stage used discrete cosine transform. Gray-wolf optimization-based PCNN models, window-based feature quality measuring filter, and singular value decomposition approach are used for fusion.	AANLIB	visual information fidelity, mutual information, peak signal-to-noise ratio, structural similarity index, entropy.	manual process, sensitive to noise, require manual parameter tuning, dictionary learning overhead, computationally heavy.
Xia et al.^17^,	Conventional (frequency domain) and sparse repre- sentation	NSST is used for decomposition. High-frequency coefficients are combined using a parameter-adaptive PCNN, whereas Low-frequency coefficients are combined using a convolutional sparse representation.	AANLIB	Entropy, Mutual information, standard deviation, average gradient, edge information retention.	manual process, sensitive to noise, dictionary learning overhead, fails to capture intricate details, computationally heavy.
Singh and Anand^18^,	Conventional (frequency domain) and sparse repre- sentation	l1-l2 decomposition is utilized by K-SVD learning, facilitating the extraction of intricate characteristics subsequently decomposed via NSST, fusion is performed using second-order derivative filter, Maximum fusion rule, and weighted average rule. Lastly, reconstruction is performed using inverse transformation.	AANLIB	Xydeas edge index, standard deviation, entropy, feature mutual information, universal image quality index, mutual information.	Because of its computational and memory requirements, NSST is less appropriate for manual processes, real-time applications, and noise artifacts.
Pathak and Tewari^20^,	Deep learning and Conventional (frequency domain)	decomposition is carried out using SWT and the coefficients are passed to pre trained VGG-16 to extract features. Maximum fusion rule and attention based fusion rule is employed. reconstruction is done using inverse SWT.	lung dataset (TCIA)	Entropy, standard deviation, mutual information, structure similarity, mean square error, peak signal-to-noise ratio	direction limitation, computational heavy, sensitive to noise.
Nair et al.^27^,	Deep learning and conventional	Two scale decomposition breakdown images into deep and approximation components. VGG11, VGG19, and squeezenet are used for extracting features. Further fusion rule for multi- layer fusion is adopted and reassembling is performed to fuse components.	AANLIB	Joint Entropy, entropy, fusion symmetry, standard deviation, edge strength, fusion factor, peak signal to noise ratio, root mean square error, spatial frequency.	registration is not performed, preprocessing is missing, Multi scale feature missing, manual process for fusion
Xia et al.^21^,	Deep learning and conventional	Gauss-Laplace and Gaussian filters are used to break down the source images, and then a deep neural network trained using a HeK-based technique is used. Prior to reconstruction, largest local variance and weighted average fusion rule is employed to fuse the high- and low-frequency components.	AANLIB	Entropy, Mutual information, standard deviation, average gradient, uniform image quality indicator, spatial frequency.	manual process for selecting fusion rule, takes time to process.
(Liu, Chen, Peng, et al.^28,29^,)	Deep learning and conventional	Laplacian pyramid technique is used to decompose the source image. The weight maps are evaluated using CNN. Fusion is performed via image pyramid whereas reconstruction is performed using inverse Laplacian pyramid.	AANLIB	Entropy, Mutual information, gradient based metric, similarity-based metrics, visual information fidelity.	manual process is used for decomposition and for selecting fusion process. Pre-processing and registration is missing.
Liu^30^,	Deep learning	The features are extracted using a multi-scale mixed attention CNN block. The feature maps are then fused using a fusion approach based on visual saliency. Lastly, reconstruction blocks based on CNN is employed to obtain the fused image.	AANLIB	correlation coefficient, spatial frequency, sum of correlation difference, mutual information.	source images are not registered, restricted to CT and MRI, need more data to train model, incapable to capture long range dependencies.
Zhang^31^,	Deep learning	A CNN-based image fusion network that collects input image attributes using 2 layers of convolutions then combines them employing elementwise-max, min, or mean rules based on image type. final image is reconstructed from the integrated attributes, enabling end-to-end training without post-processing	ImageNet	Visual information fidelity, structural similarity, spatial frequency, average gradient.	source images are not registered, Multi scale features are missing, need more data to train model, incapable to capture long range dependencies.
Xu et al.^32^,	Deep learning	For fusion, nine convolutional layers are employed, along with a Leaky ReLu transfer function. The final convolution layer is followed by the Tanh function. Shallow features in the first six convolution layers are covered by residual connections. Features are integrated via three convolutional layers.	AANLIB	Correlation coefficient, structural similarity, sum of correlation difference, peak signal to noise ratio.	limited to sequential features, lacks in multi scale features, incapable to capture long range dependencies.
Li et al.^33^,	Unsupervised Deep learning	Extraction and reassemble of features are done using Encoder and decoder using CNN. Features are fused using 4 Residual fusion networks.	MS- COCO Dataset	Modifies fusion artifacts measure, entropy, standard deviation, sum of correlation difference, mutual information, multi-scale structural similarity.	Specific to infrared and visible images only, restricted to mainly regional features, not capable to capture long term dependencies.
Chen et al.^34^,	Unsupervised Deep learning	Encoders are designed using single convolutional module and multi scale convolutional module to extract multi scale deep features. fusion is performed using average fusion rule. At last, reconstruction is performed using decoder made up of 3 single convolutional modules.	TNO public Dataset	Xydeas edge index, entropy, visual information fidelity, standard deviation, spatial frequency, Mutual information.	Restricted to local features, and not entirely automated, specific to visible and infrared images,
Song et al.^35^,	Unsupervised Deep learning	Encoders are designed using 2 convolutional layers followed by Rest2net block. Fusion is performed using spatial l1 and average attention fusion rule. At last, reconstruction is performed using decoder made up of 4 convolutional layers.	MS- COCO Dataset	Structural similarity, entropy, feature mutual information, multi-scale structural similarity, sum of correlation difference, mutual information.	Need large amount of training data, designed for natural images. restricted to regional information, manual intervention is still existed in process.
Li et al.^36^,	Unsupervised Deep learning	Multiscale deep features are extracted via encoder. spatial and channel model is used to fuse characteristics on every scale. Lastly, decoder based on nest connection is used to reconstruct the fused image.	MS- COCO Dataset	Feature mutual information, Entropy, modified structural similarity, standard deviation, mutual information, visual information fidelity.	Data intensive, Manual fusion rule is applied, computational heavy, limited to local characteristic of images, not capable to capture long term dependencies, trained on natural images.

## Methodologies

### Experimental and validation dataset

This study amalgamated two freely accessible medical imaging datasets for experimentation. The initial dataset, The Whole Brain Atlas (Keith A. Johnson & J. Alex Becker^45^,), is supplied by Harvard Medical School and comprises brain scans depicting a range of neurological illnesses, including brain images with no anomaly, brain stroke, subacute stroke, Alzheimer’s disease, and other disorders. This data repository comprises 680 brain images in various modalities, including MRI, SPECT, PET, and CT. The supplemental data repository, Lung-PET-CT-Dx (Li^46^,) is sourced from The Cancer Imaging Archive (TCIA). It comprises 342 lungs images obtained using two modalities: PET and CT. The DICOM images contain tumor annotations in the associated XML files. The PET scans have a resolution of 200 × 200, whereas the CT images are acquired at a resolution of 512 × 512. These DICOM images are converted to PNG format for streamlined processing and analysis. Extensive pre-processing processes are conducted in order to organize the data sets for the procedure of fusion. The source images are scaled and enhanced via contrast-limited adaptive histogram equalization (CLAHE) for improved visualization.

The conversion of DICOM to PNG is based on pixel normalization to the 0–255 range. Although such a change can change the absolute intensity scale, such as the Hounsfield Units (HU) in CT and Standardized Uptake Values (SUV) in PET, the main purpose of this study is to maintain the structural and textural quality of the fused image but not to investigate the quantitative intensity. The fusion framework will maintain the relative intensity distributions and anatomical structures, which are the clinically important characteristics of visual diagnosis. CLAHE is used using a clip limit of 2.0 and a tile grid of size 8 × 8 to increase the local contrast and not over-react to noise. This preprocessing step is embraced by the medical image fusion literature (^2^ Singh, Singh, Bueno, et al.^25^,) to enhance the visibility of the fine anatomical features of the image when different modalities possess a different dynamic range. Remarkably, the identical preprocessing pipeline is applied uniformly to every source image and every baseline method, so that a reasonable comparison can be made. The next generation of work will be on the development of intensity-preserving fusion pipelines which can work directly on DICOM data to maintain clinically useful intensity values to be used in quantitative diagnostic processes.

Alignment is crucial to prevent mis-registration, accomplished by decreasing the square of the distance among the source images, thereby ensuring precise alignment in terms of scales, rotation, and translation. This alignment phase is crucial for achieving regular and dependable fusion outcomes, especially for prospective medical examination applications. The Image Data Generator function is employed for image augmentation to address the constraints of a limited medical dataset. The augmentation transformations included horizontal flipping, shear transformation (10%), magnification (± 10%), rotation (± 20°), and width and height adjustments (± 10% of the total image dimensions). In order to preserve the structural integrity of the medical images, nearest-neighbor interpolation is employed. Every augmentation parameter was chosen empirically in order to generate notable variances while maintaining the essential anatomical characteristics.

### Architecture design

The proposed structure incorporates a combination of CNN and Vision Transformer^22^ to achieve medical image fusion. The novel MSML-Dense Block aimed to capture intricate features across several scales through the use of dense connections. It integrates the benefits of dense block structure with the capacity to encompass multi-scale Characteristics across Many levels, employing three Dense Blocks at distinct scales for enhanced feature extraction. The Swin Transformer, recognized for its hierarchical architecture and shifting window approach, is utilized to effectively capture global context^47^. It proficiently manages long- range dependencies and improves the feature extraction process, especially for global image attributes, so augmenting the local emphasis of the MSML Dense Block.

Algorithm 1 covers the entire MSML-DenseXmer fusion model process. There are two main steps in it. The medical dataset training comes first, followed by the fusion procedure. In order to incorporate pertinent features, two input images are first analyzed for feature extraction and then fused using an attention-based row-column vector dimension rule. After that, a convolutional layer with a tanh activation function is used to reconstruct the output into its original structure.

**Algorithm 1:**
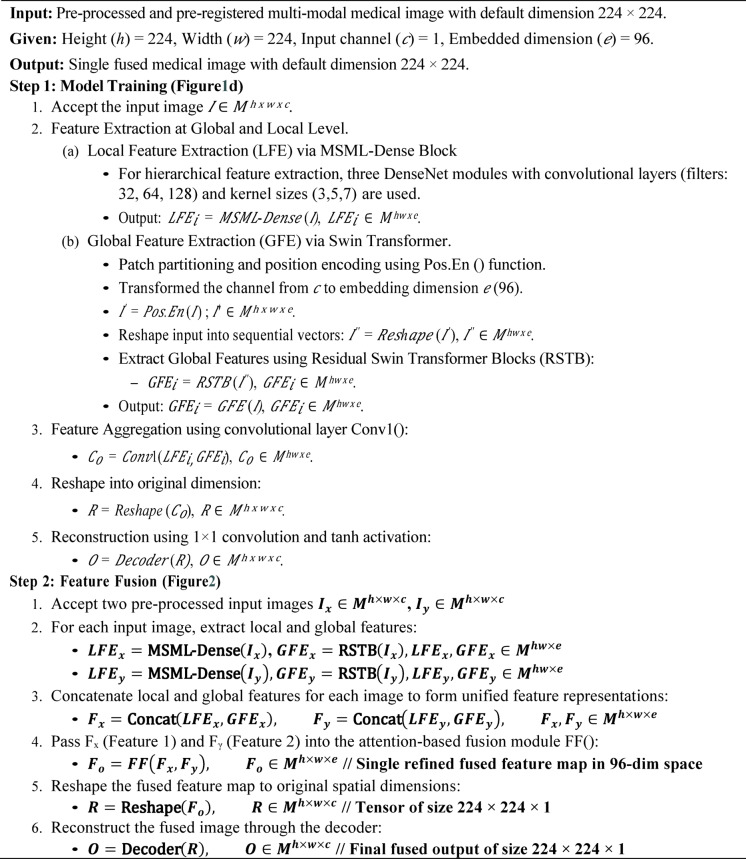
MSML-DenseXmer Fusion Model.

**Fig. 1:**
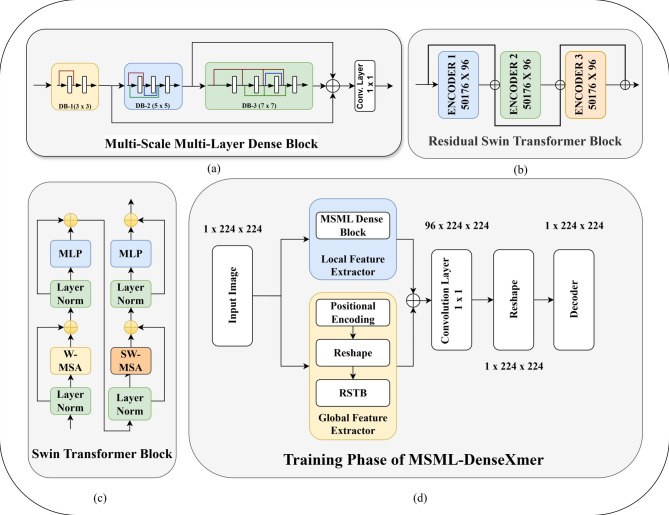
Major components of training phase of MSML-DenseXmer.

Figure 1(a-d) illustrates the internal components necessary for conducting the training of the MSML-DenseXmer model. The MSML-Dense Block, Residual Swin Transformer Block (RSTB), Swin Transformer (ST) Block, and training phase of MSML-DenseXmer are the structure’s main components.

**MSML-dense block**: Figure 1a illustrates the MSML -Dense Block design. The proposed MSML-Dense module combines multi-scale convolutional steps with dense connections to efficiently extract and merge hierarchical feature representations. Three sequential Dense blocks with increased size of kernels (3, 5, and 7) are employed to capture fine-grained, contextual, and global features. Each dense block consists of multiple convolution layers and the concatenation of all previous feature maps is fed into each layer. The output of the dense block with l^th^ layer is defined in Eq. 1 as.

1 $$\:LF{E}_{l}={H}_{l}\left(\left[{d}_{0},{d}_{1},\dots\:,{d}_{l-1}\right]\right)$$

where H_l_ ([•]) is a composite function, [•] represents channel-wise concatenation. H_l_ ([•]) is combining convolution, batch normalization and ReLU activation function. The dense connection improves parameter efficiency, mitigates the vanishing gradient problem, and reuses the features. The rationale for introducing various kernel sizes (3, 5, and 7) with increasing filter counts (32, 64, and 128) is to capture multi-scale features. Let d_1_, d_2_ and d_3_ be the outputs of three dense blocks. The feature maps are concatenated along with channel dimension to integrate multi-scale features as shown in Eq. 2.

2 $$\:LF{E}_{\mathrm{feature}}=\oplus\:\left({d}_{1},{d}_{2},{d}_{3}\right)$$

The aggregated feature map is passed through 1 × 1 convolutional layer to perform channel-wise integration and dimensionality reduction as depicted in Eq. 3.

3 $$\:LF{E}_{\mathrm{fused}}={W}_{1\times\:1}\otimes\:{d}_{\mathrm{feature}}$$

By eliminating duplication in the concatenated representation and adaptively weighing contributions from various scales, the 1 × 1 convolution serves as a learnable feature selection method. Representation is improved by fusing tiny and complex features with hierarchical ones.

**RSTB block**: Figure 1b shows RSTB structure. The system has three residually coupled encoders. The encoders work together to collect features across various stages, ensuring global feature retrieval from the original image. Each encoder block has six sequential Swin Transformer Layers (STL) with residual connections for further feature extraction. Empirical analysis determined the number of encoders and STLs needed for best output quality. The RSTB improves and collects global features (GFE), utilizing residual connections to prevent data loss during transformation. This approach ensures the model effectively captures long-range and global properties across the image, improving visuals and fusion quality.

**STL block**: As shown in Fig. 1c, each STL has 2 successive ST blocks to help the model extract more complex global characteristics. This structure provides rich global information that improves image integration precision. A Multi-Layer Perceptron (MLP) follows a Weighted Multi-Head Self- Attention (W-MSA) or Shifted Window MSA mechanism in the ST block. A Layer Normalization (LN) layer precedes each MSA and MLP component in order to normalize input. After each module, residual connections are added to ensure information integrity and training stability.

Figure 1d illustrates the procedure used in the training phase as outlined in step 1 of Algorithm 1 of the MSML-DenseXmer fusion framework. This stage covers the training process of the medical images includes the extraction and reconstruction of the input image.

**Fig. 2:**
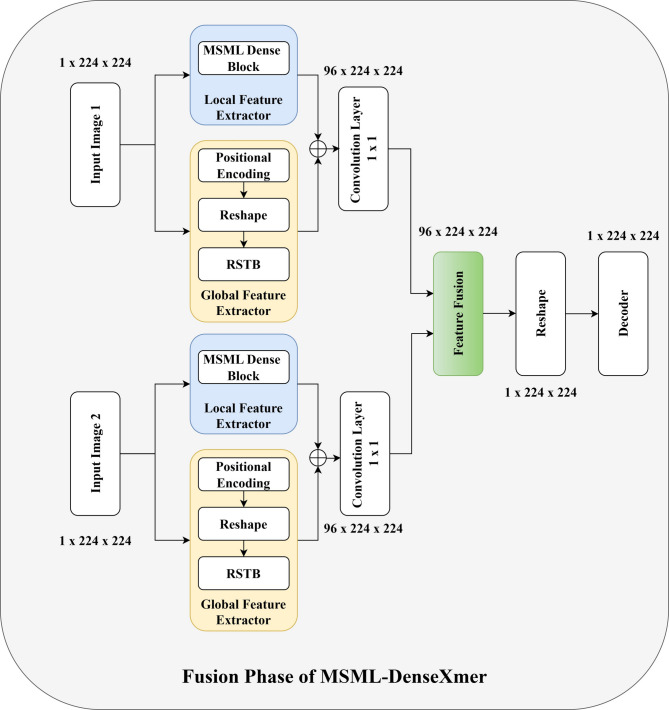
Fusion phase of MSML-DenseXmer.

Figure 2 illustrates the fusion phase of the MSML-DenseXmer fusion model (Step 2 of Algorithm 1). The detailed description of this phase, including the feature concatenation, attention-based fusion, and reconstruction process, is provided in Sect. 3.4.

**Fig. 3:**
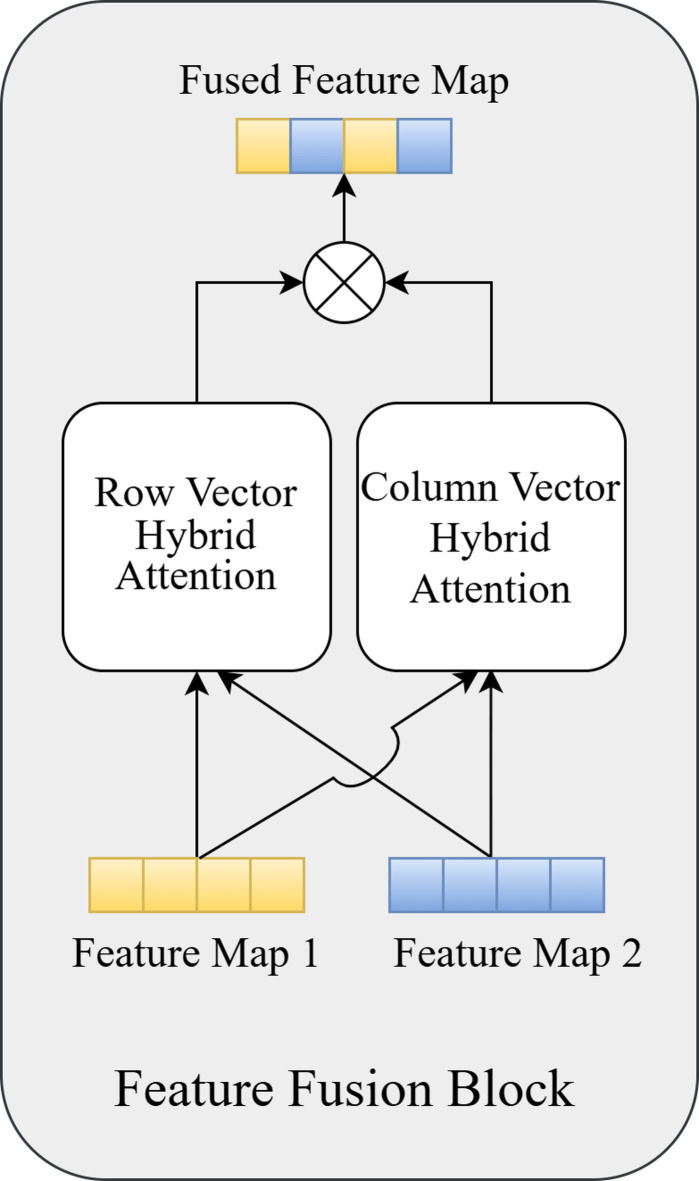
Feature fusion block.

This Block is an attention-based fusion component that integrates images utilizing a strategy based on both column and row vector dimensions. Figure 3 illustrates the architecture, demonstrating the functionality of this block. In Fig. 3, feature maps from both the input images are given to the dual dimension fusion architecture as stated in step 2 of Algorithm 1. Row vector and column vector-based attention is evaluated using a novel hybrid attention technique to attach the weights with the input tensors as given by Eqs. 4–7 and Eqs. 8–11 respectively.

4 $$\:{R}_{k}^{c}=\frac{1}{hw}|{F}_{k}^{c}{|}_{p}$$

where, c is number of channels, k is input tensors, p is type of norm

5 $$\:{R}_{k}^{c}\:=\:\alpha\:{\left|{F}_{k}^{c}\right|}_{\infty\:}\:+\:\beta\:{\left|{F}_{k}^{c}\right|}_{l2}\:+\:\gamma\:{\left|{F}_{k}^{c}\right|}_{l1},\hspace{1em}\alpha\:+\beta\:+\gamma\:=1$$

6 $$\:{W}_{r}^{x}\left(c\right)=\frac{{e}^{{R}_{r}^{x}}}{{e}^{{R}_{r}^{x}}+{e}^{{R}_{r}^{y}}},\:{W}_{r}^{y}\left(c\right)=\frac{{e}^{{R}_{r}^{y}}}{{e}^{{R}_{r}^{x}}+{e}^{{R}_{r}^{y}}}\:,\:{W}_{r}^{k}\left(c\right)\in\:{R}^{h\times\:w\times\:c}$$

7 $$\:{F}_{r}=\left({W}_{r}^{x}\odot\:{F}^{x}\right)+\left({W}_{r}^{y}\odot\:{F}^{y}\right)$$

8 $$\:{C}_{k}\left(i,j\right)=\frac{1}{c}|{F}_{k}\left(:,i,j\right){|}_{p}$$

9 $$\:({C}_{k}\left(i,j\right)=\alpha\:|{F}_{k}\left(:,i,j\right){|}_{\infty\:}+\beta\:|{F}_{k}\left(:,i,j\right){|}_{l2}+\gamma\:|{F}_{k}\left(:,i,j\right){|}_{l1})$$

10 $$\:{W}_{c}^{x}\left(i,j\right)=\frac{{e}^{{C}^{x}\left(i,j\right)}}{{e}^{{C}^{x}\left(i,j\right)}+{e}^{{C}^{y}\left(i,j\right)}},\:{W}_{c}^{y}\left(i,j\right)=\frac{{e}^{{C}^{y}\left(i,j\right)}}{{e}^{{C}^{x}\left(i,j\right)}+{e}^{{C}^{y}\left(i,j\right)}}$$

11 $$\:{F}_{C}=\left({W}_{C}^{x}\odot\:{F}^{x}\right)+\left({W}_{C}^{y}\odot\:{F}^{y}\right)$$

The novel attention is a hybrid of L1 norm, L2 norm, and infinity norm that combines the complementary characteristics of all three as referred in Eqs. 5 and 9. L1 captures distribution of intensity, L2 focuses on energy of features and infinity norm emphasizes salient features. All the norms are combined allowing the model to work with heterogeneous features.,, are the constants that balances the contribution of all the norms and provide an adaptive attention mechanism. The sum of the constants is 1 to ensure stability and their balanced contribution. The values are empirically selected to achieve the optimal feature based on heterogeneous characteristics of norm spaces. The optimal values for this study are 0.15, 0.05 and 0.8 respectively. These attention weights with respect to rows and columns are combined with input tensors as mentioned in Eqs. 7 and 11 and the element-wise sum is performed to achieve the final fused output as shown in Eq. 12.

12 $$\:{F}_{o}={F}_{r}+{F}_{C}$$

### Experimental protocols

#### Experiment1: comparison of MSML-DenseXmer with previous methods

The performance of MSML-DenseXmer fusion model is validated through a comparative analysis with conventional approaches and deep learning-based methods. The evaluation utilizes key performance metrics to compare the accuracy of all of them, thereby demonstrating the efficacy of proposed framework.

#### Experiment 2: comparison between various attention mechanism

The MSML-DenseXmer model’s performance is validated through a comparison of various attention mechanisms within the fusion rule. This experiment demonstrated the effectiveness of the hybrid attention approach compared to others. The hybrid attention approach demonstrates improved accuracy compared to individual norm-based methods.

#### Experiment 3: performance consistency among different modalities

The MSML-DenseXmer system is validated for its performance by interpreting various pairs of input images with different modalities. The output demonstrated consistent performance across various imaging modalities.

### Optimization strategy

The MSML-DenseXmer underwent training on two separate datasets featuring several modalities of medical imaging. The model utilizes the Adam optimizer and a hybrid loss function that combines pixel loss, SSIM loss, and GLCM loss to improve the effectiveness of the fusion process.

The model operates under a self-reconstruction paradigm during the training stage (Step 1 of Algorithm 1. One medical image is given as input and local features using the MSML-Dense Block and the global features using Swin Transformer are obtained. The output obtained after reconstruction is next compared to the original image, and the loss elements (MAE, SSIM and GLCM) are calculated by means of Loss (Reconstructed Output, Original input). This self-supervised training approach allows the encoder to acquire the detailed and discriminative feature representation without the need of having the ground-truth fused images, which are unavailable in nature in the medical image fusion task. The input to the fusion stage (Step 2 of Algorithm 1) is two source images, representing different modalities. Local features are calculated using the MSML-Dense Block, and global features are extracted using the Swin Transformer, simultaneously, for each image. To each input image, the local and global feature maps are concatenated to make one unified high-level feature representation which retains local and global features. These two resulting feature maps, referred to as Feature 1 and Feature 2 are then inputted into the attention-based fusion module which uses hybrid L1-L2-Infinity norm-based row-column vector dimension technique. The module produces a single refined fused feature map that is a 96-dimensional feature space. This fused feature map is reshaped back to the original spatial dimensions and turned into a 224 × 224 × 1 size tensor. The reshaped feature map is passed to the decoder that gradually recovers the final fused output image with a size of 224 × 224 × 1. The fusion phase does not make use of a loss function, because there is no ground-truth fused image. The design rationale of the framework solves the train-test objective mismatch between self-reconstruction (training) and dual-image fusion (testing). The training of self-reconstruction forces the encoder to learn fine-grained local features and its global structural information in a rich modality-specific representation. During fusion, these well-trained feature extractors operate on each source image independently, producing high-quality feature maps. These complementary features are then adaptively fused by the attention-based fusion module. As the decoder is designed to reconstruct high-fidelity images in the 96-dimensional feature space, it can faithfully reconstruct a visually coherent fused image when fused representation is in the learned feature manifold. The concatenation of local and the global features prior to fusion ensures that the fused feature map retains structural completeness as expected by the decoder.

The principal objective function is articulated as a weighted summing of these loss aspects:

13 $$Loss_{{T~}} = Loss_{{MAE}} ~ + a * Loss_{{SSIM}} ~ + a * Loss_{{GLCM}}$$

**Fig. 4:**
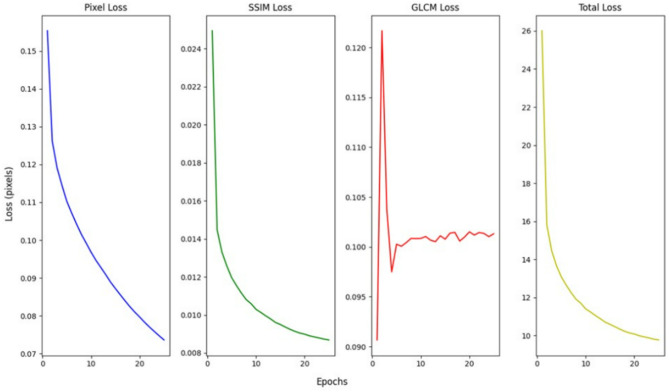
Training loss curves of the MSML-DenseXmer model across 25 epochs.

In Eq. 13, Loss_T_ denotes total loss, Loss_MAE_ signifies mean absolute error loss, Loss_SSIM_ indicates SSIM Loss and Loss_GLCM_ refers to GLCM loss. and are the weights corresponding to SSIM loss and GLCM loss, respectively. The weighting factors are employed to equilibrate the contribution of each loss component. It functions as a tuner and its value is determined empirically. In this study, the value of α and β are equal, i.e. 10^3^. This weighting factor is high since the SSIM and GLCM loss components generate values at a much smaller scale than the MAE loss. The average loss of the MAE is between 0.07 and 0.15, and the average loss of the SSIM is between 0.007 and 0.022 and the average loss of the GLCM is between 0.052 and 0.070 as shown in the training curves in Fig. 4. In the absence of scaling factor, the SSIM and GLCM components would add insignificantly to the gradient updates in the backpropagation process effectively simplifying the training process to that of just MAE-only optimization. The weight of 10^3^ is a scale-balancing factor, which makes all three components of the losses play non- trivial roles in the updates of the parameters. All the weighting parameters (1, 10, 10^2^, 10^3^, and 10^4^) were systematically tested to determine the best value. The highest weight of 10^3^ was the best performing with all quality measures of the final fused output, with 10^4^ resulting in training instability because of excessive focus on structural and textural loss at the cost of pixel-level accuracy.

The Mean Absolute Error (MAE) loss, sometimes referred to as pixel loss, quantifies the intensity disparity between the generated image and the source image. It encourages the intensity distribution as well as brightness of the merged output to closely correspond with that of the source image, preserving pixel-level precision in the fusion procedure. Equation 14 delineates the concept of MAE, also referred to as pixel loss, as follows:

14 $$Loss_{{MAE}} ~ = \frac{1}{{hw}}|F.O - I|$$

In Eq. 14, ‘h’ and ‘w’ denotes height and width, respectively, while ‘FO’ represents the fused output results and ‘I’ indicates the source image. SSIM loss evaluates the structural information of fused and reference images, thereby promoting the preservation of critical anatomical structures. This is especially important for preserving rich perceptual quality across various modalities. The SSIM loss emphasizes structural similarity, encouraging the combined image to preserve critical structural details from the source images. The SSIM loss is quantitatively expressed in Eq. 15 as follows:

15 $$Loss_{{SSIM}} ~ = 1{-}SSIM(F.O - I)$$

GLCM loss is an effective method to capture complex information on the texture of the tissue by investigating the spatial relationship of pixel pairs in a picture. GLCM currently is built by counting the number of occurrences of specific intensity values (i, j) at a given spatial separation (dx, dy) in the image. The GLCM is calculated under an offset of (1, 0) in this work indicating the horizontally neighboring pairs of pixels, hence fine-grained textural patterns in the horizontal direction are captured. GLCM loss is calculated using Eq. 16 and it is defined as the absolute difference between the GLCM of the reconstructed output and the GLCM of original source image summed over all indices of intensity pairs (i, j). This formulation punishes the deviation in the co-occurrence statistics of the reconstructed and source image and as such, the model maintains the spatial texture distribution in the reconstruction. The GLCM loss ensures that diagnostically relevant textural information including tissue homogeneity, contrast patterns and micro-structural abnormalities are effectively retained which are of critical significance in identifying aberrations in medical images.

16 $$\:{Loss}_{GLCM}=\sum\:_{i,j}\left|{GLCM}_{fused}\left(i,j\right)-\:{GLCM}_{source}(i,j)\:\right|$$

The integrated loss function balances critical aspects to improve fused medical images. Pixel Loss preserves borders and textures, but SSIM Loss retains structural similarity for large-scale anatomical characteristics. Detail tissue examination requires precise textural patterns preserved by GLCM Loss. These losses guarantee the model balances luminance accuracy, perceptual excellence, and textural integrity for high-quality fused images. This hybrid technique improves the suggested fusion framework for several medical imaging modalities.

### Implementation details

The fusion of medical scans of different modalities is done by the MSML-DenseXmer. In this study, two publicly available health care imaging databases were combined to experiment with, which led to a total of 5,589 clinical images of various modalities. This includes different combinations of the brain imaging modalities: MRI-T2 plus SPECT, MRI-T1 plus PET, MRI-T2 plus PET, MRI-T1 plus CT, MRI-T2 plus CT, and MRI-T1 plus SPECT, and CT-PET images relevant to lung diseases. The MSML-DenseXmer model is trained with Adam optimizer with the following settings: learning rate=1e-5, β₁=0.9, β_2_ = 0.999, ε = 1e-8. The learning rate remains constant during training without learning rate scheduling since the low initial learning rate of 1e-5 ensures stable and consistent convergence and consistent in all the epochs. The model is learned under a self-reconstruction paradigm, which aims at reconstructing the input image based on extracted features. The total population of 5,589 augmented medical images with all possible modality combinations is stratified into an 80:20 ratio with 80% (4,471 images) of the population being used to train the encoder-decoder network and 20% (1,118 images) to serve as a validation set to track the quality of reconstruction and indicate overfitting. The final model is that model that has the least validation loss. The quality of fusions is not compared with any ground-truth fused images, so at the fusion stage, never-before-seen pairs of multi-modal images are fused and evaluated by the quantitative measures as outlined in Sect. 5. This assessment plan is in line with the previously existing unsupervised fusion models like DenseFuse and U2Fusion. The individual and total loss curves over 25 training epochs are plotted in Fig. 4.

The pixel loss (MAE) and SSIM loss differing curves have a gradual and consistent decrease which means that the model is gradually learning to reconstruct input images with increased precision and structural fidelity at pixels level. The GLCM loss exhibits a rapid decrease at the first few epochs then a stabilization, indicating that the model adapts to textural patterns fast, then fine-tunes in subsequent epochs. The total loss curve exhibits a consistent downward trend, with the loss decreasing from approximately 26 to 9 and stabilizing beyond epoch 20. Additional training was attempted (up to 50 epochs) but this did not show any improvements, indicating that 25 epochs is the best time to stop.

Table 2 shows all the architectural and training hyperparameters of the MSML-DenseXmer framework. The best values are determined by systematic exploration within the ranges listed, and the fusion quality and computational efficiency are prioritized.

**Table 2:** Hyperparameters used in MSML-DenseXmer.

S.No.	Hyperparameter	Optimal values	Experimental value
1	Batch size	4	4,8,16
2	Patch size	1	1, 2, 3, 4
3	Window size	7	4,7,8
4	Learning rate	1e-5	1e-4, 1e-3, 1e-2
5	Epoch	25	10, 15, 20, 25, 30, 35, 40, 45, and 50
6	Image size	224 × 224	224 × 224
7	Input channel	1	Gray scale image
8	Output channel	1	Gray scale image
9	Embedding dimension	96	48, 96, 192
10	Number of attention heads	3	1, 2, 3, 4, 6
11	MLP ratio	4	2, 3, 4
12	Number of RSTB blocks	3	1, 2, 3, 4, 5
13	Number of STL per RSTB	6	2, 4, 6, 8
14	Number of ST blocks per STL	2	1, 2, 3
15	MSML-Dense: Growth Rate	32	16, 32, 64

The number of trainable parameters and floating-point operations (FLOPs) are measured to describe the computation complexity of the proposed framework. MSML-DenseXmer model has about 6.52 million trainable parameters, including the MSML-Dense Block (1.82 M), the Swin Transformer backbone (4.21 M) as well as the feature aggregation, fusion, and decoder modules (0.49 M). A single forward pass of the model on an input image of 224 × 224 × 1 would cost around 18.42 GFLOPs. A comparative analysis between model complexity and the benchmark methods is provided in Table 3.

**Table 3:** Comparative analysis of model complexity.

Model	Trainable parameters (M)	FLOPs (G)	Fusion time (s)	Peak GPU memory (GB)
DWT-BL	N/A (non-learnable)	N/A	0.42	N/A
IFCNN	0.08	0.56	0.02	0.31
DenseFuse	0.07	0.51	0.55	0.28
U2Fusion	0.66	4.83	0.53	0.92
MSML-DenseXmer	6.52	18.42	2.46	3.74

As indicated in Table 3, the MSML-DenseXmer model has a larger parameter count and computational cost compared to the baseline methods. This increased complexity is attributable to the dual-branch architecture comprising both the MSML-Dense Block and the Swin Transformer, which are essential for the simultaneous extraction of local and global features. The Swin Transformer backbone accounts for approximately 64.6% of the total parameters owing to the multi-head self-attention mechanism across 36 transformer blocks. However, the window-based attention mechanism reduces the computational complexity from quadratic to linear with respect to image size, thereby ensuring scalability.

The training procedure is conducted using a hardware configuration comprising eight A100 NVIDIA GPU cards at JIIT, Noida, India. To evaluate the model’s feasibility on standard hardware, inference is also performed on a single NVIDIA T4 GPU (16 GB), which is freely accessible through platforms such as Google Colab. Table 4 presents the runtime comparison across both hardware configurations. The MSML-DenseXmer achieves a fusion time of approximately 5.83 s per image pair on the T4 GPU with a peak memory consumption of 3.18 GB, which is well within the 16 GB capacity of the T4. This confirms that the model does not require specialized high-end hardware for deployment and can operate on widely accessible GPUs.

**Table 4:** Runtime analysis on different hardware configurations.

Model	Hardware	GPU memory (Total)	Fusion time per pair (s)	Peak GPU usage (GB)
DWT-BL	CPU (no GPU required)	N/A	0.42	N/A
IFCNN	1× NVIDIA T4	16 GB	0.04	0.12
DenseFuse	1× NVIDIA T4	16 GB	0.89	0.11
U2Fusion	1× NVIDIA T4	16 GB	0.87	0.58
MSML-DenseXmer	1× NVIDIA T4	16 GB	5.83	3.18
MSML-DenseXmer	8× NVIDIA A100	80 GB each	2.46	3.18

Although the fusion time is higher compared to lightweight CNN-based methods such as IFCNN (0.02s), it remains acceptable for offline clinical diagnostic workflows where fusion quality is prioritized over real-time processing. For resource-constrained or real-time deployment scenarios, the model can be further optimized through knowledge distillation, model pruning, or reducing the number of RSTB blocks, enabling a configurable trade-off between computational efficiency and fusion quality. The optimization of the model is achieved by integrating mean absolute error, structural similarity loss, and GLCM loss functions. The source code and pre-trained models will be made publicly available upon acceptance of this manuscript.

### Performance evaluation

This section assesses the proposed MSML-DenseXmer system through recognized qualitative as well as quantitative metrics. It outlines the applied metrics, displays results in a table-like structure, and includes graphical illustrations of the merged outputs for the brain as well as lung images.

### Performance evaluation metrics (PEM)

This study employed various qualitative metrics represented in Table 5 to assess the quality of single fused scans. Entropy (EN) quantifies the information content in an image, with greater entropy signifying increased detail captured during the fusion process^48^. Spatial frequency (SF) evaluates the degree of textural details, essential for recognizing intricate patterns in medical imaging^49^. Mean Square Error (MSE) and Root Mean Square Error (RMSE) quantify pixel-wise discrepancies between the fused and reference images, thereby ensuring minimal variation in intensity distribution^50^. Structural Similarity Index (SSIM) and Multi-Scale SSIM (MSSIM) assess structural consistency, promoting the retention of anatomical features throughout the fusion process^51^. Feature similarity (FSIM) denotes the resemblance in features^52^. High FSIM demonstrated more pertinent characteristics. NMI (Normalized Mutual Information) quantifies the shared information between input and fused images, thereby ensuring content relevance^53^. VIF (Visual Information Fidelity) measures visual quality, promoting the perceptual quality of the merged output^54^. Finally, PSNR (Peak Signal-to-Noise Ratio) evaluates the general degree of quality by contrasting the original and fused images, where higher PSNR values signify improved quality and reduced distortion^50^. These metrics collectively offer a thorough assessment of the fused images regarding specifics, structure, and perceptual excellence, thereby confirming the efficacy of the fusion procedure across various dimensions.

**Table 5:** Performance indicator metrics.

SN	PEM	Expressions in mathematics
1	EN	EN = −$\:{\sum\:}_{\mathrm{i\:=\:0}}^{\mathrm{L-1}}p\left(i\right){\mathrm{log}}_{2}p\left(i\right)$;where p(i) is the probability of occurrence of intensity i in the image.
2	SF	SF= $\:\sqrt{{RF}^{2}+{CF}^{2}}$,where RF =$\:\sqrt{\frac{1}{m(n-1)}{\sum\:}_{i=1}^{m}{\sum\:}_{j=2}^{n}{\left(x\left(i,j-1\right)-x(i,j)\right)}^{2}}$ CF = $\:\sqrt{\frac{1}{n(m-1)}{\sum\:}_{i=2}^{m}{\sum\:}_{j=1}^{n}{\left(x\left(i,j\right)-x(i-1,j)\right)}^{2}}$ where m= rows, n = columns, x(i, j) is the intensity value at i^th^ row and j^th^ column pixel.
3	SSIM	SSIM (x, y) = $\:\frac{\left(2\mu\:x\:\mu\:y+c1\left)\right(2\sigma\:xy+c2\right)}{({\mu\:x}^{2\:}+{\mu\:y}^{2}+c1)\:\left({\sigma\:x}^{2}+\:{\sigma\:y}^{2}+c2\right)}$; where $\:{\mu\:}_{x}$ is the sample mean, $\:{{\upsigma\:}}_{\mathrm{x}}^{2}\:$ is the variance and $\:{{\upsigma\:}}_{\mathrm{x}\mathrm{y}}$ is the covariance of image x and image, c1 and c2 are two constants.
4	MSE	MSE =$\:\frac{1}{mn}{\sum\:}_{i=1}^{m}{\sum\:}_{j=1}^{n}{\left(FO\left(i,j\right)-S(i,j)\right)}^{2}$; where FO (i, j) is a fused output and S (i, j) is a reference image.
5	MSSIM	MSSIM= $\:\frac{1}{k}{\sum\:}_{i=1}^{k}SSIM({FO}_{i}$ |$\:{S}_{i}$); where FO is a fused output and S is a source image, k is the number of local windows in the image.
6	RMSE	RMSE = $\:\sqrt{\frac{1}{mn}{\sum\:}_{i=1}^{m}{\sum\:}_{j=1}^{n}{\left(FO\left(i,j\right)-S(i,j)\right)}^{2}}$; where m= rows, n = columns, FO (i, j) is a fused output image and S (i, j) is a reference image.
7	PSNR	PSNR = 10*$\:{\mathrm{log}}_{10}\left(\frac{{MAX}_{i}^{2}}{MSE}\right)$; where MAX is the maximum intensity value of an image.
8	NMI	NMI = $\:\frac{2\:*\:MI(FO;S)}{EN\left(FO\right)+EN\left(S\right)}$, where FO is a fused image and S is a source image, MI is the mutual information *\:MI(FO;S)* = EN (FO)–EN (FO | S); where EN is an entropy.
9	VIF	VIF = $\:\frac{{\sum\:}_{k}{\sum\:}_{b}{\mathrm{log}}_{2}\left(1+\:\frac{{g}_{k,b\:}^{2}.{\left({\sigma\:}_{k,b}^{r}\right)}^{2}}{\left({\left({\sigma\:}_{k,b}^{d}\right)}^{2}-\:{g}_{k,b\:}^{2}{\left({\sigma\:}_{k,b}^{r}\right)}^{2}+\:{\sigma\:}_{n}^{2}\right)}\right)}{{\sum\:}_{k}{\sum\:}_{b}{\mathrm{log}}_{2}\left(1+\:\frac{{\left({\sigma\:}_{k,b}^{r}\right)}^{2}}{{\sigma\:}_{n}^{2}}\right)}$; where $\:{\sigma\:}_{k,b}^{r,d}\:$ is the covariance of source image and output image in the b^th^ block at the kth sub-band, $\:{g}_{k,b\:}^{.}$ is the scalar gain field.
10	FSIM	FSIM = $\:\frac{{\sum\:}_{x\in\:\varOmega\:}{S}_{L}\left(x\right)*\:{PC}_{m}\left(x\right)}{{\sum\:}_{x\in\:\varOmega\:}{PC}_{m}\left(x\right)}$;S_L_ is similarity at location x; PC_m_ is maximum phase congruency.

### Qualitative output

**Fig. 5:**
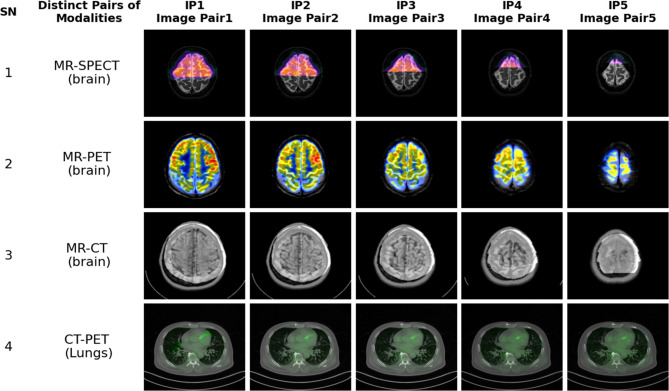
Visual representation of fused images from MRI, PET, SPECT, and CT modalities using MSML-DenseXmer.

Figure 5 illustrates the qualitative results of the proposed MSML-DenseXmer framework, displaying the fused scans from four various modalities pairs: MRI-PET, MRI-SPECT, MRI-CT, and CT-PET. Each pair illustrates the model’s capacity to integrate images from diverse modalities while maintaining both the structural and textural integrity. The combined outputs demonstrate improved visual quality, clearly depicting both high-intensity areas and intricate anatomical details. This illustrates the efficacy of the suggested framework in extracting pertinent information from several modalities, hence producing a balanced and relevant fused image with potential applicability for subsequent clinical evaluation.

### Quantitative output

The quantitative results, displayed in tabular style, provide a thorough array of metrics to assess the efficacy of the fusion approach across five pairs of images from various modality groups, including MRI-PET, MRI-SPECT, MRI- CT, and CT-PET. The 10 mentioned metrics jointly evaluate the quality of the fused images, encompassing contrast, structural integrity, error reduction, and perceptual accuracy. The Table 6 illustrates that the MSML-DenseXmer architecture consistently achieves improved performance across several modalities, producing high-quality fusion results.

**Table 6:** Quantitative outcomes for fused scans from various modality combinations.

Distinct	EN	SSIM	SF	MSE	PSNR	NMI	RMSE	MSSIM	FSIM	VIF
Pairs of modalities										
MRI-SPECT (brain)	2.2158	0.8875	5.7393	0.0064	70.0535	2.6470	0.0802	0.8717	0.9481	0.8859
MRI-PET (brain)	2.8861	0.8521	7.4021	0.0141	67.0419	2.6075	0.1162	0.8445	0.9276	0.6881
MRI-CT (brain)	2.7036	0.8474	8.2601	0.0327	62.9952	2.6543	0.1807	0.7495	0.8838	0.7087
CT-PET (Lungs)	5.6589	0.7551	15.8471	0.0167	65.9555	2.2718	0.1289	0.6523	0.7136	0.7246

## Results and discussion

### Comparison with previous works

The initial experiment evaluates the efficacy of the MSML-DenseXmer in comparison to several baseline models, encompassing conventional models, and cutting-edge deep learning fusion techniques. The benchmarking methods include both conventional and state-of-the-art deep learning approaches: DWT-BL^3^, IFCNN^31^, DenseFuse^55^, U2Fusion^32^, SwinFusion^56^, and CDDFuse^57^. SwinFusion employs a pure Swin Transformer architecture with cross-domain fusion, while CDDFuse utilizes a dual-branch decomposition network with correlation-driven fusion. These recent methods are included to provide a comprehensive comparison against the latest transformer-based and decomposition-based fusion approaches.

The baseline methods are DWT-BL^3^, which uses DWT decomposition, bilateral filtering to weight coefficients; IFCNN^31^, a CNN-based approach that uses convolutional layers to extract features, use summation/maximization to fuse, and reconstruct features; DenseFuse^55^ which uses an encoder-decoder architecture with dense blocks and L1-norm based feature fusion; and U2Fusion^32^, which employs nine convolutional layers with residual connections. Although these techniques capture local features effectively, they suffer from a common shortcoming: they cannot capture long-range context and global structure, in addition to fine-grained local textures, which limits their fusion quality across a range of medical imaging techniques.

Traditional and deep learning approaches to medical image fusion struggle to simultaneously capture long-range dependencies, detailed textures and complex global structures, leading to an insufficient representation of features. These limitations highlight their regional feature biases. CNNs excel at capturing local textures but fall short of capturing global features, while transformer-based methods focus on capturing large amounts of contextual information, but may ignore the detailed textures and deep anatomical variance, resulting in poor fusion quality. To address these challenges, the MSML-DenseXmer paradigm combines the benefits of Swin Transformer and CNN. It uses Swin-Transformer to capture global features and MSML dense block to extract multi-scale and multi-level regional characteristics. Also, the attention-based fusion technique is applied, which results in improved integration of both local and global information and hence contributes to better performance across all measures. Table 7 presents the performance of the MSML-DenseXmer in comparison to four benchmark frameworks, assessed using ten distinct criteria. It also shows the time required by the models to fuse a pair of input images in seconds. The model’s mean metric error is calculated by first computing the error for each metric for all input images, and then aggregating these values to get the overall error.

The percentage improvement over each baseline method is calculated as given in Eq. 17:

17 $$\:\mathrm{\%\:Increase}=\left(\frac{{E}_{\mathrm{baseline}}-{E}_{\mathrm{proposed}}}{{E}_{\mathrm{baseline}}}\right)\times\:100$$

This measure illustrates how well various methods integrate various medical imaging modalities. The MSML-DenseXmer model is noteworthy for its consistent reliability and strong fusion performance across a range of medical image modalities. The results are consistent across 10 evaluation metrics. Despite the fact that MSML-DenseXmer has a higher image fusion time for a pair of input images (2.46 s) than other methods, it uses the MSML-Dense Block and Swin Transformer to combine local and global features effectively. Our approach focuses on preserving features and quality of fusion as they are essential for medical image fusion applications. But it consistently outperforms existing techniques in quantitative and qualitative evaluation. Moreover, the use of window attention in the transformer allows better scalability of the model with reduced computation compared to global attention methods. The model can also be fine-tuned for deployment by adjusting the architecture parameters such as depth, embedding size and attention heads to strike a balance between efficiency and performance.

**Table 7:** Comparison of mean metric error among benchmark and MSML-DenseXmer fusion models.

Mean metric error	MRI-SPECT	MRI_PET	MRI-CT	Lungs	Fusion time (seconds)
DWT-BL	15.66	13.93	20.65	25.95	0.42
% Increase wrt proposed model	46.16	40.13	61.26	64.28	
IFCNN	9.74	9.14	10.7	10.59	0.02
% Increase wrt proposed model	13.44	8.75	25.23	12.46	
DenseFuse	9.42	8.98	9.8	10.16	0.55
% Increase wrt proposed model	10.51	7.13	18.37	8.76	
U2Fusion	9.36	8.95	9.92	9.54	0.53
% Increase wrt proposed model	9.94	6.82	19.35	2.83	
MSML-DenseXmer	8.43	8.34	8	9.27	2.46
SwinFusion	8.91	8.72	8.54	9.68	1.83
% Increase wrt proposed	5.39	4.36	6.32	4.23	
CDDFuse	8.78	8.63	8.41	9.52	1.92
% Increase wrt proposed	3.99	3.36	4.88	2.63	
MSML-DenseXmer	8.43	8.34	8	9.27	2.46

### Comparison between various attention mechanism

In this study, we used a range of attention-based methods for feature fusion using row and column vector spaces. The attention methods explored are comprised of the L1-norm, L2-norm, and Infinity-norm, as well as combinations of these norms with different properties. The L1-norm enhances relevant features and reduces the influence of less important ones, enabling a focus on key features. The L2-norm treats all features uniformly, but is more susceptible to outliers. The infinity-norm focuses on the most important features but tends to ignore the less important ones. Combination of these norms enables us to use the best of both worlds.

The L1L2INF and three other combinations of normalization techniques are compared. The constants α, β, γ are best set to their values of 0.15, 0.05 and 0.8 respectively performing better than the others. This allows to collect strong and weak features and thus leads to balanced and precise features combination. The results shown in Table 8 demonstrate that the L1L2INF approach has the lowest error and achieves the best accuracy among all the approaches, hence proving its efficacy over the data set of brain visual images.

To further analyze the sensitivity of the hybrid attention mechanism to the weighting constants α, β, and γ, a systematic evaluation is conducted by varying their values while maintaining the constraint α + β + γ = 1. Table 9 shows the mean metric error on all the metrics on the brain MRI-SPECT data set for various weights. The weight of infinity norm (γ) ranges from 0.5 to 0.9, with the other two weights (α and β) being adjusted accordingly.

**Table 8:** Evaluation Of L1, L2, AND Infinity norm combinations for enhanced fusion quality.

Fusion with Attention	EN	SSIM	SF	MSE	PSNR	NMI	RMSE	MSSIM	FSIM	VIF	Mean metric error
L1	1.9819	0.9003	5.5027	0.0063	70.3627	2.7127	0.0784	0.8799	0.9518	0.8871	8.4264
L2	1.9825	0.9003	5.5026	0.0063	70.3607	2.7128	0.0784	0.8799	0.9518	0.8865	8.4262
INF	1.9849	0.8992	5.3353	0.0065	70.5006	2.6635	0.0800	0.8793	0.9520	0.8818	8.4183
L1L2	1.9856	0.8998	5.4186	0.0062	70.4347	2.6833	0.0776	0.8796	0.9519	0.8851	8.4222
L1_INF	1.9856	0.8998	5.4185	0.0062	70.4363	2.6831	0.0776	0.8796	0.9519	0.8854	8.4224
L2_INF	1.9822	0.9003	5.5022	0.0063	70.3618	2.7129	0.0784	0.8799	0.9518	0.8868	8.4263
L1L2INF	1.9863	0.8996	5.3511	0.0060	70.4893	2.6675	0.0701	0.8594	0.9520	0.8825	8.4164

**Table 9:** Sensitivity analysis of attention weight constants (α, β, γ) for L1L2INF Hybrid Attention.

Configuration	α (L1)	β (L2)	γ (INF)	EN	SSIM	SF	MSE	PSNR	RMSE	Mean metric error
C1	0.33	0.33	0.34	1.9831	0.8997	5.4412	0.0064	70.3215	0.0789	8.4249
C2	0.25	0.25	0.5	1.984	0.8995	5.4105	0.0063	70.3846	0.0781	8.4221
C3	0.2	0.1	0.7	1.9851	0.8996	5.3788	0.0062	70.4352	0.0753	8.4196
C4	0.15	0.05	0.8	**1.9863**	**0.8996**	**5.3511**	**0.006**	**70.4893**	**0.0701**	**8.4164**
C5	0.1	0	0.9	1.9855	0.8993	5.3396	0.0063	70.4471	0.0778	8.4185
C6	0.4	0.1	0.5	1.9836	0.8998	5.4298	0.0063	70.3518	0.0785	8.4238
C7	0.05	0.15	0.8	1.9858	0.8994	5.3603	0.0061	70.4681	0.0724	8.4178

The sensitivity analysis presents many key observations. The first and the most important point to be noticed is the performance of the model is highly sensitive to γ. As the γ increases from 0.34 to 0.8, the mean metric error consistently decreases, indicating that emphasizing salient features through the γ is critical for fusion quality. Secondly, if γ is slightly more increased to 0.9, the model performance degrades, clearly suggesting that completely suppressing the L1 and L2 contributions removes useful information about intensity. Third, on comparing C4 and C7 configuration, it was observed that the model performs marginally better when L1 (intensity distribution) receives a higher weight than L2 (feature energy), which aligns with the importance of preserving intensity patterns in medical image fusion. Lastly, the optimal configuration C4 the lowest mean metric error of 8.4164, confirming that a dominant infinity norm with a secondary L1 contribution and a minor L2 contribution provides the most balanced and effective attention weighting for multi-modal medical image fusion.

### Performance consistency among different modalities

The proposed MSML-DenseXmer system is thoroughly evaluated to determine its performance with various combinations of medical imaging modalities. The proposed framework is carefully tested using various combinations of MRI, CT, PET and SPECT scans to assess its effectiveness and flexibility with multi-dataset types. The system is also validated with lung image data as well as brain image data, showing it can swiftly adapt and yield competently across a range of medical imaging conditions.

**Fig. 6:**
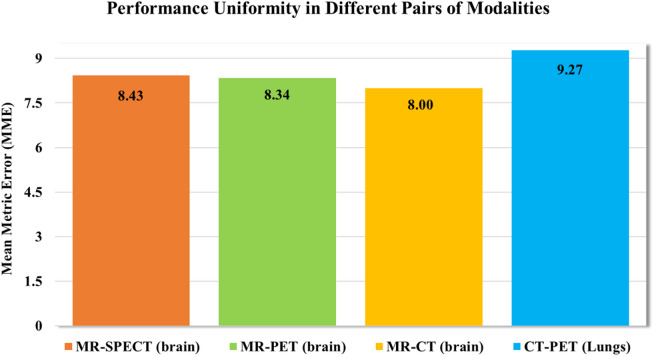
Cross-modality based performance of the presented framework on brain and lungs images.

As shown in Fig. 6, the model demonstrates minimal mean metric error with brain and lung data, thus maintaining its performance over all possible combinations of imaging modalities. The study demonstrates the ability of the model to offer accurate and reliable fusion of different medical images. Accuracy and reliability are crucial for medical imaging applications, where the reliable fusion of different types of data is required for diagnosis. The model’s success across various datasets and imaging modalities demonstrates its usefulness, and shows its ability to perform in a generalized environment.

### Ablation studies

An ablation test is performed on the MRI-SPECT brain dataset to better understand the role of each key component. The study compares the following five model variants: (A1) only the MSML-Dense Block to extract features, (A2) only the Swin Transformer, (A3) train the whole architecture with MAE loss, (A4) train with MAE and SSIM losses but without GLCM loss, and (A5) the entire MSML-DenseXmer model. The results are shown in Table 10.

**Table 10:** Ablation study on MRI-SPECT brain dataset.

Variant	Component	EN	SSIM	SF	MSE	PSNR	RMSE	Mean metric error
A1	MSML-Dense only	1.9214	0.8812	5.1847	0.0081	69.0423	0.0901	8.6831
A2	Swin only	1.9508	0.8903	5.0126	0.0074	69.4218	0.0862	8.5947
A3	Full model + MAE only	1.9625	0.8841	5.2194	0.0072	69.6135	0.0847	8.5412
A4	Full model + MAE + SSIM	1.9781	0.8962	5.2918	0.0066	70.1247	0.0812	8.4536
A5	**MSML-DenseXmer (Full)**	**1.9863**	**0.8996**	**5.3511**	**0.006**	**70.4893**	**0.0701**	**8.4164**

The ablation results in Table 10 show the impact of each component. The combination of local and global feature extraction (A5) outperforms MSML-Dense-only (A1) and Swin-only (A2) variants by 3.07% and 2.07% respectively, reduces the mean metric error, validating the synergy between local and global features. The Swin-only model (A2) achieves better results than the MSML-Dense-only model (A1), suggesting that global features are slightly more important to fusion quality. But the MSML-Dense Block supplements the Swin Transformer with local textural detail, leading to a further increase in performance.

The loss function ablation (A3-5) shows the contributions of each loss part. The inclusion of SSIM loss (A3 → A4) leads to a decrease of the mean metric error by 1.02%, thereby validating the importance of preserving the structural integrity of the image for enhancing the fusion quality. The inclusion of GLCM loss (A4 → A5) leads to another 0.44% improvement, confirming that direct preservation of texture information is an important component of fusion quality. Although the improvement with GLCM is modest, it’s essential for preserving tissue textures that are important for diagnosis but cannot be captured by pixel-level and structural losses.

### Downstream task evaluation

To demonstrate the potential use of the fused images, a lung tumor segmentation task is conducted using a pretrained U-Net. The Dice Similarity Coefficient (DSC) on the Lung-PET-CT-Dx dataset is used to assess the segmentation accuracy. Table 11 below shows the segmentation accuracy with individual modality and fused images.

**Table 11:** Downstream lung tumor segmentation performance using individual modality and fused images on the lung-PET-CT-Dx dataset.

Input	Dice score	Precision	Recall
CT only	0.7214	0.7452	0.7103
PET only	0.6538	0.6821	0.6347
Fused (MSML-DenseXmer)	0.7863	0.8012	0.7741
Fused (U2Fusion)	0.7521	0.7684	0.7398
Fused (DenseFuse)	0.7438	0.7612	0.7295

Table 11 shows that fusion-based segmentations consistently outperform segmentation with single modality images. The MSML-DenseXmer fused images attain a Dice score of 0.7863, a 9.0% increase compared to segmentation using only CT and a 20.3% boost with respect to only PET. Moreover, fused images generated by the proposed framework result in improved segmentation accuracy over fused images generated by U2Fusion and DenseFuse, showing that the high-quality fused images lead to improved performance in downstream clinical tasks.

## Conclusion

In this study, we propose the MSML-DenseXmer system to tackle the issue of multimodal medical image fusion. The MSML-DenseXmer framework enhances the quality of the image using novel components, such as the MSML Dense Block and Residual Swin Transformer Block, that address the problem of extracting local and global features. The system employs a novel combination of loss functions, including pixel loss, SSIM loss and GLCM loss, to improve the model’s performance by maintaining an accurate distribution of intensities, structure and textural information of the fused output images. The use of different attention mechanisms, including L1, L2 and Infinity norms, aids in the image fusion process, balancing the attention between prominent and weak features. The comparison with state-of-the-art baseline models suggests that the proposed MSML-DenseXmer framework results in better accuracy with reduced error in different medical image datasets, such as brain and lungs images. The use of a variety of quantitative and qualitative metrics confirms the efficacy of the MSML-DenseXmer model in different applications. These results suggest that the proposed model has potential for clinical applications, pending further validation through downstream clinical tasks such as segmentation and classification studies. Future work will focus on extending the structure to a wider range of modalities and exploring improvements in feature extraction and fusion approaches. This study advances the field of medical image processing by offering a framework enhancing the quality and visualization of medical images.

## Data Availability

The data used in this research is publicly accessible.
